# Serum microRNAs as new criteria for referral to early palliative care services in treatment-naïve advanced cancer patients

**DOI:** 10.18632/oncotarget.28327

**Published:** 2022-12-17

**Authors:** Tomofumi Miura, Shuichi Mitsunaga, Juntaro Matsuzaki, Satoko Takizawa, Ken Kato, Atsushi Ochiai, Takahiro Ochiya

**Affiliations:** ^1^Division of Biomarker Discovery, Exploratory Oncology Research and Clinical Trial Center, National Cancer Center Hospital East, Kashiwa, Japan; ^2^Department of Palliative Medicine, National Cancer Center Hospital East, Kashiwa, Japan; ^3^Department of Hepatobiliary and Pancreatic Oncology, National Cancer Center Hospital East, Kashiwa, Japan; ^4^Division of Molecular and Cellular Medicine, National Cancer Center Research Institute, Tokyo, Japan; ^5^Division of Pharmacotherapeutics, Keio University Faculty of Pharmacy, Tokyo, Japan; ^6^Toray Industries, Inc., Kamakura, Japan; ^7^Department of Gastrointestinal Medical Oncology, National Cancer Center Hospital, Tokyo, Japan; ^8^Pathology Division, Exploratory Oncology Research and Clinical Trial Center, National Cancer Center Hospital East, Kashiwa, Japan; ^9^Department of Molecular and Cellular Medicine, Tokyo Medical University, Tokyo, Japan

**Keywords:** microRNA, early palliative care, integration, cancer, referral

## Abstract

A major obstacle to the implementation of early palliative care (EPC) is the lack of objective criteria for referral to EPC. Circulating microRNAs (miRNAs) have been recognized as promising biomarkers. The present study investigated objective definitions for referral to EPC using microRNA. A total of 178 serum samples were obtained from patients with lung, gastrointestinal, colorectal, bile duct, pancreas and bladder cancers who were treatment-naïve and received chemotherapy between January 2011 and December 2013 at National Cancer Center Hospital East. We investigated expression levels of miRNAs using microarrays. The primary outcome was prediction of admission to a palliative care unit ≤6 months after first visit. Diagnostic models using clinical characteristics, miRNAs and combinations of both were constructed. The miRNA models were constructed using 6 miRNA levels. The best areas under the receiver operating characteristic curve (AUCs) of the clinical model was 0.741, while the average AUCs of miRNA-based models and combination models were 0.769 and 0.806, respectively. Combination models showed higher AUCs than the clinical model (*p* < 0.023). The present combination models might offer new objective definitions for referral to EPC and thus contribute to real-world implementation of EPC.

## INTRODUCTION

The importance of early palliative care (EPC) has gained attention after the 2010 study by Temel et al. [1]. EPC has been shown to improve symptom management and quality of life, increase psychological well-being and satisfaction among caregivers, and reduce aggressive end-of-life care in several randomized controlled studies [2, 3]. The lack of human resources in specialty palliative care services and ambiguities in the indications for referral to palliative care have inhibited the social implementation of EPC. Palliative care specialists have proposed indications for referral to palliative care on the basis of the Delphi study of palliative care specialists from all over the world [4]. However, these indications lacked objective variables, including results from blood testing. Easy, useful, objective indices are thus needed to facilitate the implementation of EPC.

Tumor markers, tumor staging system and performance status (PS) are common objective prognostic indices in patients with treatment-naïve advanced cancer. As the underlying disease progresses, physical functions deteriorate in several ways, including persistently severe disability, progressive disability throughout the course, catastrophic disability within 1–3 months before death, or potentially no disability in the event of sudden death [5]. Identification of patients in whom physical function will deteriorate soon after the start of anti-tumor treatment might be more important than identification of short life expectancy for the purpose of implementing EPC, because EPC includes advanced care planning, management of physical symptoms and psychological distress, and support for the living in their desired places [6]. Our previous study showed the 80% of patients newly admitted to the palliative care unit (PCU) had a Eastern Cooperative Oncology Group - performance status (ECOG-PS) of 3 or 4 [7]. We therefore considered that identification of patients who will be rapidly admitted to the PCU at the start of anti-tumor treatment is crucial to defining indications for referral to EPC.

MicroRNA (miRNA) is small, non-coding RNA, 20–25 nucleotides in length. The function of miRNA is to regulate gene expression through suppressing the translation of target genes or degrading target mRNAs [8]. Furthermore, extracellular miRNAs play important roles in intercellular communication [9]. Serum miRNAs have recently been recognized as prognostic indices in gastrointestinal cancer [10], lung cancer [11], pancreatic cancer [12], and hepatocellular carcinoma [13]. However, no miRNA studies have investigated the prediction of early deterioration of physical function in advanced cancer patients. Several miRNAs from muscle (myomiRs) have been shown to be involved in muscle degradation in lung cancer patients [14]. Serum miRNAs may thus be applicable to predicting early deterioration of physical function in patients with advanced cancers.

The aim of the present study was to develop predictive models using serum miRNAs for patients who admitted to a PCU ≤6 months after starting anti-tumor treatment.

## RESULTS

A total of 178 subjects (mean age, 64.3 + 9.7 years; female, 34.9%) were enrolled in this study (Table 1). The three most common types of cancer were lung cancer (60.7%), pancreas cancer (17.5%), and stomach cancer (9.0%). Of these, 74.8% of subjects were Stage IV. Patients admitted to a PCU in ≤6 months (early deterioration group) and >6 months (non-deterioration group) comprised 16.3% and 83.8%, respectively.

**Table 1:** Background characteristics of patients

Variables	Category	*n*	(%)
Age	Mean (SD)	64.3	(9.7)
	<65 years	82	(46.1)
	>65 years	96	(54.0)
Sex	Male	116	(65.2)
	Female	62	(34.9)
Primary site	Stomach	16	(9.0)
	Esophagus	9	(5.1)
	Colorectal	7	(4.0)
	Biliary tract	6	(3.4)
	Lung	108	(60.7)
	Bladder	1	(0.6)
	Pancreas	31	(17.5)
Stage	2	12	(6.8)
	3	33	(18.6)
	4	133	(74.8)
ECOG-PS at diagnosis	0	94	(52.9)
	1	78	(43.9)
	2	6	(3.4)
BMI	Mean (SD)	22	(3.0)
	<22.0 kg/m^2^	92	(51.7)
	>22.0 kg/m^2^	86	(48.4)

Abbreviations: PS: performance status; BMI: body mass index; PCU: palliative care unit.

### Construction of the clinical predictive model

First, we selected candidate variables using univariate analysis (Table 2). PS, serum concentrations of lactate dehydrogenase (LDH) and C-reactive protein (CRP), and neutrophil count (Neut) were significantly higher in the early deterioration group than in the non-deterioration group. Conversely, serum concentrations of albumin (Alb) and hemoglobin (Hb) were significantly lower in the deterioration group than in non-deterioration group.

**Table 2:** Differences between patient characteristics and interval to palliative care unit admission

			>6 months (*n* = 149)	<6 months (*n* = 29)	*p*-value
Age	<65 years	*n* (%)	70	(47.0)	12	(41.4)	0.580
	>65 years	*n* (%)	79	(53.0)	17	(58.6)	
Sex	Male	*n* (%)	96	(64.4)	20	(69.0)	0.639
	Female	*n* (%)	53	(35.6)	9	(31.0)	
Primary site	Stomach	*n* (%)	12	(8.1)	4	(13.8)	0.137
	Esophagus	*n* (%)	8	(5.4)	1	(3.5)	
	Colorectal	*n* (%)	6	(4.0)	1	(3.5)	
	Biliary tract	*n* (%)	5	(3.4)	1	(3.5)	
	Lung	*n* (%)	95	(63.8)	13	(44.8)	
	Bladder	*n* (%)	0	(0)	1	(3.5)	
	Pancreas	*n* (%)	23	(15.4)	8	(27.6)	
PS	0	*n* (%)	83	(55.7)	11	(37.9)	0.031^*^
	1	*n* (%)	63	(42.3)	15	(51.7)	
	2	*n* (%)	3	(2.0)	3	(10.3)	
Stage	2	*n* (%)	11	(7.4)	1	(3.5)	0.127
	3	*n* (%)	31	(20.8)	2	(6.9)	
	4	*n* (%)	107	(71.8)	26	(89.7)	
BMI	<22.0 kg/m^2^	*n* (%)	74	(49.7)	18	(62.1)	0.221
	>22.0 kg/m^2^	*n* (%)	75	(50.3)	11	(37.9)	
Alb		Mean (SD)	4.2	(0.4)	3.7	(0.6)	<.0001^*^
AST		Mean (SD)	28.3	(36.1)	33.8	(30.8)	0.446
ALT		Mean (SD)	32.9	(78.9)	27.1	(20.4)	0.695
ChE		Mean (SD)	255	(89.3)	237.4	(105)	0.417
LDH		Mean (SD)	241.7	(171.6)	408.4	(579.9)	0.004^*^
T-Bil		Mean (SD)	1.00	(2.3)	0.9	(1)	0.905
BUN		Mean (SD)	13.8	(4.4)	14.9	(6.7)	0.234
Cre		Mean (SD)	0.7	(0.2)	0.8	(0.2)	0.923
Na		Mean (SD)	139.6	(2.9)	138.5	(5)	0.099
K		Mean (SD)	4.2	(0.3)	4.2	(0.5)	0.716
Cl		Mean (SD)	102.6	(3.3)	102	(4.3)	0.383
RBC		Mean (SD)	437.7	(48.4)	417.5	(60.3)	0.051
Hb		Mean (SD)	13.5	(1.6)	12.5	(1.9)	0.004^*^
Plt		Mean (SD)	25.4	(8.7)	24.3	(8)	0.533
CRP		Mean (SD)	1.6	(2.8)	3.7	(5.6)	0.002^*^
Neut		Mean (SD)	5142.1	(2054.2)	6699.7	(5207)	0.007^*^
Lymp		Mean (SD)	1688.3	(532.1)	1534.5	(617.9)	0.167

Abbreviations: PS: performance status; BMI: body mass index; Alb: albumin; AST: asparate aminotransferase; ALT: alanine aminotransferase; BUN: blood urea nitrogen; Hb: hemoglobin; Plt: platelet; CRP: C-reactive protein; Neut: neutrophil count; Lymp: lymphocyte count.

The clinical predictive model was constructed as: diagnostic index = (−7.819) + (1.819) × (Alb) + (7.503 × 10^−2^) × (CRP) + (2.558 × 10^−3^) × (PS) + (−1.237 × 10^−3^) × (LDH) + (2.193 × 10^−1^) × (Hb) + (−7.428 × 10^−5^) × (Neut) (Supplementary Table 1). The receiver operating characteristic (ROC) curve of this clinical predictive model showed and area under the ROC curve (AUC) of 0.741, with sensitivity of 0.448 and specificity of 0.973 (Table 3).

In addition, the diagnostic ability of each clinical variable in the above clinical predictive model are shown in Supplementary Table 2.

**Table 3:** Diagnostic ability of each prognostic model

	AUC	Sensitivity	Specificity	PPV	NPV
Clin model	0.741	0.448	0.973	0.765	0.901
miR-model-01	0.764	0.966	0.604	0.322	0.989
miR-model-02	0.765	0.966	0.591	0.315	0.989
miR-model-03	0.735	0.862	0.557	0.275	0.954
miR-model-04	0.735	0.897	0.584	0.295	0.967
miR-model-05	0.735	0.862	0.611	0.301	0.958
miR-model-06	0.765	0.966	0.624	0.333	0.989
miR-model-07	0.730	0.828	0.664	0.324	0.952
miR-model-08	0.736	0.931	0.557	0.290	0.976
miR-model-09	0.769	0.966	0.617	0.329	0.989
miR-model-10	0.742	0.828	0.611	0.293	0.948
Comb-model-01	0.802	0.828	0.624	0.300	0.949
Comb-model-02	0.802	0.828	0.624	0.300	0.949
Comb-model-03	0.806	0.862	0.591	0.291	0.957
Comb-model-04	0.797	0.919	0.517	0.556	0.907
Comb-model-05	0.797	0.919	0.517	0.556	0.907
Comb-model-06	0.800	0.839	0.621	0.429	0.919
Comb-model-07	0.806	1.000	0.450	0.261	1.000
Comb-model-08	0.799	0.966	0.477	0.264	0.986
Comb-model-09	0.804	0.793	0.658	0.311	0.942
Comb-model-10	0.806	0.828	0.617	0.296	0.948

Abbreviations: Alb: albumin; CRP: C-reactive protein; PS: performance status; Hb: hemoglobin; Neut: neutrophil count; AUC: area under the curve; PPV: positive predictive value; NPV: negative predictive value. ^*^Compared to Clin model using DeLong Test.

### Construction of miRNA predictive models

Cross-validation by logistic LASSO regression analysis was performed to identify the optimal combination of miRNAs for predicting early deterioration. The mean AUC of 10 candidate models according to the number of miRNA probes reached a ceiling with 6 miRNA probes (Supplementary Figure 1). We therefore listed the top 10 predictive miRNA models using 6 miRNA levels (Supplementary Table 1). AUCs for each model ranged from 0.730 to 0.769 (Table 3).

In addition, the diagnostic ability of each miRNA in these miRNA predictive models is shown in Supplementary Table 2.

### Construction of combined clinical characteristic and miRNA probe models

**Figure 1:**
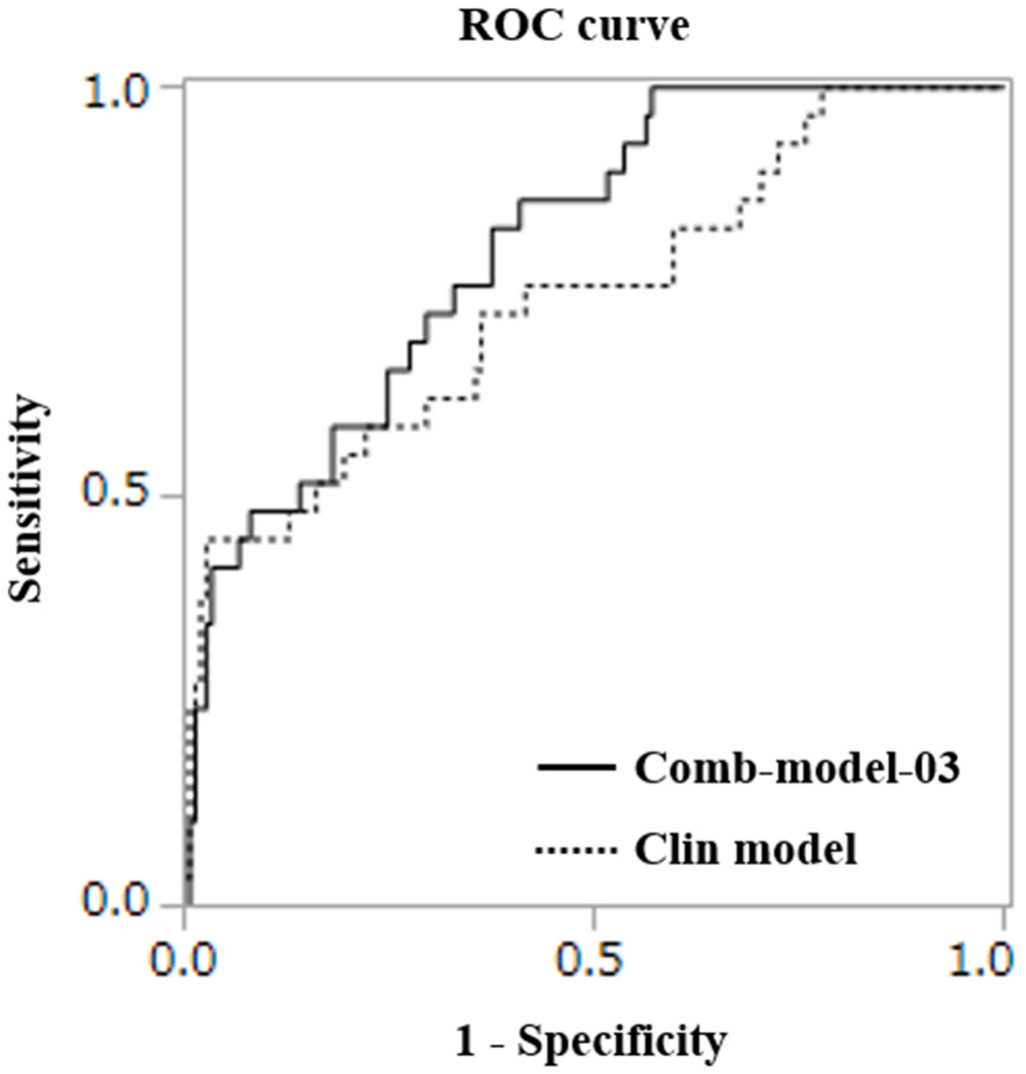
ROC curves for detecting the early deterioration group.

We constructed models combining the clinical model and miRNA models. Details of the 10 combination models are shown in Supplementary Table 1. The best combination model was constructed as follows: diagnostic index = (−17.330) + (−1.814 × 10^−1^) × (miR-486-5p) + (3.245 × 10^−1^) × (miR-7846-3p) + (−6.517 × 10^−1^) × (miR-642b-3p) + (4.123 × 10^−1^) × (miR-4707-5p) + (1.120) × (miR-3663-3p) + (−1.900 × 10^−1^) × (miR-4732-5p) + (1.693) × (Alb) + (1.084 × 10^−1^) × (CRP) + (3.319 × 10^−2^) × (PS) + (−9.630 × 10^−4^) × (LDH) + (1.824 × 10^−1^) × (Hb) + (−8.891 × 10^−5^) × (Neut). The AUC for the best combination model was 0.806, with sensitivity of 0.862 and specificity of 0.591. All AUCs in combination models were higher than that in the clinical model (Figure 1, Table 3).

## DISCUSSION

The present study developed objective prediction models using miRNAs and clinical indices for patients admitted to a PCU ≤6 months after starting anti-tumor treatment. No serum miRNAs showed significant changes in expression level or were associated with poor prognosis, and were associated with previously reported myomiRs.

The most important finding was the development of an objective prediction model for patients admitted to a PCU within 6 months among treatment-naïve advanced cancer patients. The present objective prediction model might be recognized as one criterion for EPC and therefore might contribute to oncologists identifying patients for referral to EPC. The lack of definitive criteria for referral to EPC is one of the most important obstacles to the implementation of EPC [15]. Objective indices in previous Delphi studies for palliative care specialists have included only spinal cord compression [4]. Other criteria such as life expectancy, symptoms, distress and delirium involved some degree of subjectivity and were sometimes difficult to diagnose. Such subjectivity might confuse oncologists and present a barrier to referral to EPC. Another observational study revealed that a system triggering automatic referral to palliative care according to definitions including some objective criteria for performance status and cancer type increased referrals for palliative care consultation [16]. That study might suggest the importance of objective criteria. In addition, the physical function of advanced cancer patients [17] not only deteriorates rapidly within a few months before death, but also deteriorates in several different manners, including persistent severe, slowly progressive, and rapidly progressive before death, or no decline before death [5]. The present study tried to develop an objective prediction model for PCU admission within 6 months. An objective prediction model could be considered for an objective criterion for referral to EPC, and would help oncologists in identifying patients in need of referral to EPC and subsequent increases in quality of life for those patients.

The second important finding was that: (1) miRNAs known as prognostic factors or myomiRs in previous studies did not show changes in expression level or detection, and (2) a combination of miRNAs could improve the diagnostic capability for PCU admission ≤6 months after starting chemotherapy. The miRNAs known as prognostic factors include miR-760, miR-150-3p, miR-122, miR-187, miR-675 and miR-204, and are involved with cellular invasion, proliferation and vascularization [10–13]. The present study did not detect significant changes in expression levels of those miRNAs (data not shown). These results might indicate differences between the prognosis itself and the deterioration of physical function. On the other hand, previous research has shown that myomiRs regulated various functions including cell growth, differentiation, stress responsiveness and protection against apoptosis, and also correlated with muscle atrophy and muscle size [18, 19]. Changes in expression level of myomiRs were reported in muscles from patients with several cancer types, including lung cancer [14], and pancreatic and colorectal cancers [20]. However, the present study did not detect any significant changes in expression levels of the following myomiRs identified in previous studies: miRNA-424-5p, miRNA-424-3p, miRNA-450a, miRNA-451a and miRNA-144-5p, miR-3184-3p, miR-423-5p, let-7d-3p, miR-1296-5p, miR-345-5p, miR-532-5p, miR-423-3p or miR-199a-3p (data not shown). Recently, research involving a healthy aging sprinter showed that serum miR-21 and miR-146a were associated with declining physical performance [4]. The present study did not detect expression of these miRNAs. This result might indicate the myomiRs play an important function in regulating local systems. Furthermore, the present results showed that each miRNA probe had low diagnostic ability to predict physical deterioration (Supplementary Tables). Combination with miRNA probes was thus important to increasing diagnostic ability, compared to individual miRNA probes. Our predictive model might reflect systemic changes in the bodies of patients, and induce changes to the microenvironment including myocytes and myomiRs. Our preliminary analysis using miEAA (https://ccb-compute2.cs.uni-saarland.de/mieaa2/) showed that changes in expression of miRNAs were involved in increased function of CD15-, CD56-, CD19-, CD3-, and CD14-positive cells, respectively (data not shown). Myeloid-derived suppressor cells [21, 22], regulatory T cells [23], and the tumor-promoting phenotype of monocytes [24] have recently received a great deal of research attention. Such cells play immunosuppressive roles and correlate with poor prognosis. The predictive models in this present study might thus reflect immunotolerance.

The present study displayed several limitations. First, the present study was performed using retrospectively collected samples from a single center. The time from blood collection to deep freezing before microarray analysis was not strictly regulated. Other situations involving sample handling such as centrifugation, storage and storage temperature were uniformly performed. Second, we lacked an extra validation cohort to confirm any diagnostic capability of the present predictive models. We intend to prospectively assess the generalizability of our predictive models.

## MATERIALS AND METHODS

### Subjects and sample collection

Serum samples of advanced cancer patients were obtained from patients referred to the National Cancer Center Hospital East (NCCE) who were histologically diagnosed with cancer. Samples were registered to the National Cancer Center biobank between 2008 to 2016 and stored at −80°C until further use. Among these, patients who were >20 years old, with ECOG-PS ranging between 0 to 2, with gastrointestinal, lung, hepatobiliary, pancreas or bladder cancer, who were chemo-naïve at enrollment, and who had received chemotherapy at NCCE, and who were admitted to a palliative care unit (PCU) in the National Cancer Center Hospital East during 2011 to 2013 were enrolled in this study. Patients who had already received any other treatment elsewhere, whose treatment were surgery and radiotherapy, or who had synchronous or metachronous cancer were excluded. Clinical characteristics were collected from electrical medical records. Subjects were divided into two groups according to the interval between first visit and admission to the PCU ≤6 months (early deterioration group) or >6 months (non-deterioration group) after presentation.

### Construction of clinical diagnostic models

Candidate variables were selected using univariate analysis for duration for deterioration. Multivariate linear regression modeling for short deterioration were constructed. AUCs for short deterioration were determined from ROC curves.

### Serum miRNA expression analysis

Total RNA was extracted from 300 μL of serum using the 3D-Gene RNA Extraction Reagent (Toray Industries, Inc.). Comprehensive miRNA expression analysis was performed using the 3D-Gene miRNA Labeling Kit and the 3D-Gene Human miRNA Oligo Chip (Toray Industries, Inc.), which was designed to detect 2,588 miRNAs registered in miRBase release 21 (http://www.mirbase.org/ [25]). Fluorescent signals for each spot on the microarray were obtained using the 3D-Gene Microarray Scanner (Toray Industries, Inc.) and digitized using the “Extraction” accessory digitizing application (Toray Industries, Inc.). For quality control of microarray data, criteria for low-quality results were as follows: (i) coefficient of variation for negative control probes >0.15; and (ii) number of flagged probes identified as an uneven spot image by 3D-Gene Scanner >10. Samples meeting these criteria were excluded from further analyses. Presence of miRNAs was determined on the basis of a corresponding microarray signal greater than the [mean + 2′ standard deviation (SD)] of the negative control signal from which the top and bottom 5%, ranked by signal intensity, were removed. Once a miRNA was considered present, the mean signal of the negative controls was subtracted from the miRNA signal. When the signal value was negative (or undetected) after background subtraction, the value was replaced by the lowest signal intensity minus 0.1 on a base-2 logarithm scale. To normalize signals among the microarrays tested, three preselected internal control miRNAs (miR-149-3p, miR-2861, and miR-4463) were used, as described previously [19].

### Construction of diagnostic model using miRNA

The best combinations of miRNAs were explored using Fisher linear discriminant analysis with leave-one-out cross-validation [26]. Briefly, the best 10 discriminants for single miRNAs were selected, one of the residual miRNAs was added to generate two-miRNA discriminants, and the best 10 two-miRNA discriminants were selected. This method was used to generate 1–10-miRNA discriminants.

Mean AUCs of the 10 best discriminants for each number of miRNAs were calculated. Then we identified the minimal number of miRNAs to achieve the best AUC and fixed the number of miRNAs for making discriminants in further analysis. Each AUC for 10 different discriminants with the fixed number of miRNAs were calculated. In addition, diagnostic indices in both short deterioration and non-deterioration groups were also calculated.

### Combined miRNA and clinical models

Multivariate linear regression analysis for short deterioration was performed using each of the 10 miRNA discriminants and the clinical model. AUCs for each of the 10 combination models and the diagnostic index were calculated. To show improvements in diagnostic performance, the AUC for the model combining miRNAs and the clinical model and the AUC for the clinical model were compared using the DeLong test.

### Statistics

Continuous variables including laboratory data and categorical data including age <65 or >65 years, sex, BMI <22.0 or >22.0 kg/m^2^, primary cancer site, PS, and stage were calculated using the *t*-test or chi-square test, respectively. Linear discriminant analysis and model selection based on leave-one-out cross-validation were performed using R version 3.1.2 (R Foundation for Statistical Computing, http://www.R-project.org), compute.es package version 0.2-4, hash package version 2.2.6, MASS package version 7.3-45, mutoss package version 0.1-10, and pROC package version 1.8. AUCs for the construct combining miRNA discriminants and the clinical model using the DeLong test were analyzed using JMP for Windows version 12.0 (SAS Institute, Cary, NC, USA). The level of significance was set as *p* < 0.05.

## CONCLUSION

The present study developed a predictive model using miRNA for patients admitted to a PCU ≤6 months after starting anti-tumor treatment. The present models might offer objective criteria for oncologists to facilitate the referral of patients to the EPC.

## SUPPLEMENTARY MATERIALS



## References

[1] Temel JS , Greer JA , Muzikansky A , Gallagher ER , Admane S , Jackson VA , Dahlin CM , Blinderman CD , Jacobsen J , Pirl WF , Billings JA , Lynch TJ . Early palliative care for patients with metastatic non-small-cell lung cancer. N Engl J Med. 2010; 363:733–42. 10.1056/nejmoa1000678. 20818875

[2] Zimmermann C , Swami N , Krzyzanowska M , Hannon B , Leighl N , Oza A , Moore M , Rydall A , Rodin G , Tannock I , Donner A , Lo C . Early palliative care for patients with advanced cancer: a cluster-randomised controlled trial. Lancet. 2014; 383:1721–30. 10.1016/S0140-6736(13)62416-2. 24559581

[3] Temel JS , Greer JA , El-Jawahri A , Pirl WF , Park ER , Jackson VA , Back AL , Kamdar M , Jacobsen J , Chittenden EH , Rinaldi SP , Gallagher ER , Eusebio JR , et al. Effects of Early Integrated Palliative Care in Patients With Lung and GI Cancer: A Randomized Clinical Trial. J Clin Oncol. 2017; 35:834–41. 10.1200/JCO.2016.70.5046. 28029308 PMC5455686

[4] Hui D , Mori M , Watanabe SM , Caraceni A , Strasser F , Saarto T , Cherny N , Glare P , Kaasa S , Bruera E . Referral criteria for outpatient specialty palliative cancer care: an international consensus. Lancet Oncol. 2016; 17:e552–59. 10.1016/S1470-2045(16)30577-0. 27924753

[5] Gill TM , Gahbauer EA , Han L , Allore HG . Trajectories of disability in the last year of life. N Engl J Med. 2010; 362:1173–80. 10.1056/NEJMoa0909087. 20357280 PMC2877372

[6] Yoong J , Park ER , Greer JA , Jackson VA , Gallagher ER , Pirl WF , Back AL , Temel JS . Early palliative care in advanced lung cancer: a qualitative study. JAMA Intern Med. 2013; 173:283–90. 10.1001/jamainternmed.2013.1874. 23358690

[7] Miura T , Matsumoto Y , Hama T , Amano K , Tei Y , Kikuchi A , Suga A , Hisanaga T , Ishihara T , Abe M , Kaneishi K , Kawagoe S , Kuriyama T , et al. Glasgow prognostic score predicts prognosis for cancer patients in palliative settings: a subanalysis of the Japan-prognostic assessment tools validation (J-ProVal) study. Support Care Cancer. 2015; 23:3149–56. 10.1007/s00520-015-2693-x. 25777319

[8] Kim VN , Han J , Siomi MC . Biogenesis of small RNAs in animals. Nat Rev Mol Cell Biol. 2009; 10:126–39. 10.1038/nrm2632. 19165215

[9] Kim VN . MicroRNA biogenesis: coordinated cropping and dicing. Nat Rev Mol Cell Biol. 2005; 6:376–85. 10.1038/nrm1644. 15852042

[10] Zheng Q , Chen C , Guan H , Kang W , Yu C . Prognostic role of microRNAs in human gastrointestinal cancer: A systematic review and meta-analysis. Oncotarget. 2017; 8:46611–23. 10.18632/oncotarget.16679. 28402940 PMC5542297

[11] Castro D , Moreira M , Gouveia AM , Pozza DH , De Mello RA . MicroRNAs in lung cancer. Oncotarget. 2017; 8:81679–85. 10.18632/oncotarget.20955. 29113423 PMC5655318

[12] Guo S , Fesler A , Wang H , Ju J . microRNA based prognostic biomarkers in pancreatic Cancer. Biomark Res. 2018; 6:18. 10.1186/s40364-018-0131-1. 29942514 PMC5963153

[13] Zhang YC , Xu Z , Zhang TF , Wang YL . Circulating microRNAs as diagnostic and prognostic tools for hepatocellular carcinoma. World J Gastroenterol. 2015; 21:9853–62. 10.3748/wjg.v21.i34.9853. 26379392 PMC4566380

[14] van de Worp WRP , Schols AMW , Dingemans AC , Op den Kamp CMH , Degens JHR , Kelders MCJ , Coort S , Woodruff HC , Kratassiouk G , Harel-Bellan A , Theys J , van Helvoort A , Langen RCJ . Identification of microRNAs in skeletal muscle associated with lung cancer cachexia. J Cachexia Sarcopenia Muscle. 2020; 11:452–63. 10.1002/jcsm.12512. 31828982 PMC7113505

[15] Aldridge MD , Hasselaar J , Garralda E , van der Eerden M , Stevenson D , McKendrick K , Centeno C , Meier DE . Education, implementation, and policy barriers to greater integration of palliative care: A literature review. Palliat Med. 2016; 30:224–39. 10.1177/0269216315606645. 26405109

[16] Adelson K , Paris J , Horton JR , Hernandez-Tellez L , Ricks D , Morrison RS , Smith CB . Standardized Criteria for Palliative Care Consultation on a Solid Tumor Oncology Service Reduces Downstream Health Care Use. J Oncol Pract. 2017; 13:e431–40. 10.1200/jop.2016.016808. 28306372

[17] Seow H , Barbera L , Sutradhar R , Howell D , Dudgeon D , Atzema C , Liu Y , Husain A , Sussman J , Earle C . Trajectory of performance status and symptom scores for patients with cancer during the last six months of life. J Clin Oncol. 2011; 29:1151–8. 10.1200/JCO.2010.30.7173. 21300920

[18] Kovanda A , Režen T , Rogelj B . MicroRNA in skeletal muscle development, growth, atrophy, and disease. Wiley Interdiscip Rev RNA. 2014; 5:509–25. 10.1002/wrna.1227. 24838768

[19] Lee DE , Brown JL , Rosa-Caldwell ME , Blackwell TA , Perry RA Jr , Brown LA , Khatri B , Seo D , Bottje WG , Washington TA , Wiggs MP , Kong BW , Greene NP . Cancer cachexia-induced muscle atrophy: evidence for alterations in microRNAs important for muscle size. Physiol Genomics. 2017; 49:253–60. 10.1152/physiolgenomics.00006.2017. 28341621

[20] Narasimhan A , Ghosh S , Stretch C , Greiner R , Bathe OF , Baracos V , Damaraju S . Small RNAome profiling from human skeletal muscle: novel miRNAs and their targets associated with cancer cachexia. J Cachexia Sarcopenia Muscle. 2017; 8:405–16. 10.1002/jcsm.12168. 28058815 PMC5476855

[21] Gabitass RF , Annels NE , Stocken DD , Pandha HA , Middleton GW . Elevated myeloid-derived suppressor cells in pancreatic, esophageal and gastric cancer are an independent prognostic factor and are associated with significant elevation of the Th2 cytokine interleukin-13. Cancer Immunol Immunother. 2011; 60:1419–30. 10.1007/s00262-011-1028-0. 21644036 PMC3176406

[22] Diaz-Montero CM , Salem ML , Nishimura MI , Garrett-Mayer E , Cole DJ , Montero AJ . Increased circulating myeloid-derived suppressor cells correlate with clinical cancer stage, metastatic tumor burden, and doxorubicin-cyclophosphamide chemotherapy. Cancer Immunol Immunother. 2009; 58:49–59. 10.1007/s00262-008-0523-4. 18446337 PMC3401888

[23] Cheng H , Luo G , Lu Y , Jin K , Guo M , Xu J , Long J , Liu L , Yu X , Liu C . The combination of systemic inflammation-based marker NLR and circulating regulatory T cells predicts the prognosis of resectable pancreatic cancer patients. Pancreatology. 2016; 16:1080–84. 10.1016/j.pan.2016.09.007. 27665172

[24] Chittezhath M , Dhillon MK , Lim JY , Laoui D , Shalova IN , Teo YL , Chen J , Kamaraj R , Raman L , Lum J , Thamboo TP , Chiong E , Zolezzi F , et al. Molecular profiling reveals a tumor-promoting phenotype of monocytes and macrophages in human cancer progression. Immunity. 2014; 41:815–29. 10.1016/j.immuni.2014.09.014. 25453823

[25] Kozomara A , Griffiths-Jones S . miRBase: annotating high confidence microRNAs using deep sequencing data. Nucleic Acids Res. 2014; 42:D68–73. 10.1093/nar/gkt1181. 24275495 PMC3965103

[26] Yokoi A , Matsuzaki J , Yamamoto Y , Tate K , Yoneoka Y , Shimizu H , Uehara T , Ishikawa M , Takizawa S , Aoki Y , Kato K , Kato T , Ochiya T . Serum microRNA profile enables preoperative diagnosis of uterine leiomyosarcoma. Cancer Sci. 2019; 110:3718–26. 10.1111/cas.14215. 31599471 PMC6890430

