# Supplementary material for: Serum microRNAs as new criteria for referral to early palliative care services in treatment-naïve advanced cancer patients
Source: Oncotarget. 2022 Dec 17;13:1341–9. doi: 10.18632/oncotarget.28327 (PMC9760266; doi:10.18632/oncotarget.28327)
Supplement: Supplementary file 1 [file oncotarget-13-28327-s001.pdf]

Serum microRNAs as new criteria for referral to early palliative care services in treatment-naïve advanced cancer patients

SUPPLEMENTARY MATERIALS

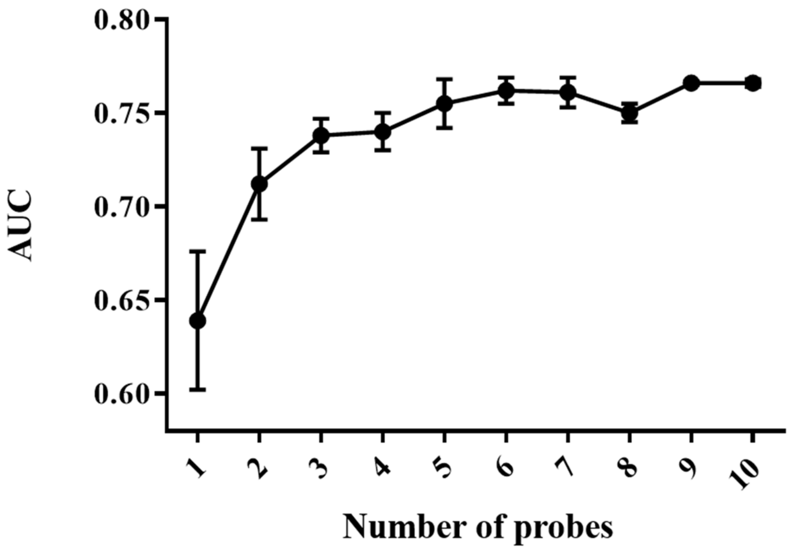

Supplementary Figure 1: Mean AUC of the 10 candidate miRNA models by the number of miR probes.

Supplementary Table 1: Details of each predictive model

	Predictive models
Clin	(−7.819) + (1.819) × (Alb) + (7.503 × 10^{-2}) × (CRP) + (2.558 × 10^{-3}) × (PS) + (−1.237 × 10^{-3}) × (LDH) + (2.193 × 10^{-1}) × (Hb) + (−7.428 × 10^{-5}) × (Neut)
miR-model-01	(−5.799) + (−1.285) × (miR-642b-3p) + (−3.785 × 10^{-1}) × (miR-6768-5p) + (−5.585 × 10^{-2}) × (miR-4758-5p) + (−4.436 × 10^{-1}) × (miR-6821-5p) + (1.513) × (miR-3663-3p) + (1.074) × (miR-7107-5p)
miR-model-02	(−8.830) + (1.084 × 10^{-1}) × (miR-3648) + (−1.327) × (miR-642b-3p) + (−5.256 × 10^{-1}) × (miR-6768-5p) + (−1.471 × 10^{-1}) × (miR-4725-3p) + (1.553) × (miR-3663-3p) + (1.052) × (miR-7107-5p)
miR-model-03	(−10.771) + (−2.162 × 10^{-1}) × (miR-486-5p) + (3.866 × 10^{-1}) × (miR-7846-3p) + (−7.765 × 10^{-1}) × (miR-642b-3p) + (4.913 × 10^{-1}) × (miR-4707-5p) + (1.334) × (miR-3663-3p) + (−2.264 × 10^{-1}) × (miR-4732-5p)
miR-model-04	(−13.476) + (−8.259 × 10^{-1}) × (miR-642b-3p) + (6.845 × 10^{-1}) × (miR-4758-5p) + (1.235) × (miR-3663-3p) + (−1.085 × 10^{-1}) × (miR-296-5p) + (5.114 × 10^{-1}) × (miR-4433b-3p) + (−2.838 × 10^{-1}) × (miR-4732-5p)
miR-model-05	(−13.935) + (−8.494 × 10^{-1}) × (miR-642b-3p) + (7.479 × 10^{-1}) × (miR-4758-5p) + (−2.568 × 10^{-2}) × (miR-8071) + (1.208) × (miR-3663-3p) + (4.937 × 10^{-1}) × (miR-4433b-3p) + (−2.830 × 10^{-1}) × (miR-4732-5p)
miR-model-06	(−8.164) + (2.251 × 10^{-1}) × (miR-4745-5p) + (−1.420) × (miR-642b-3p) + (−4.611 × 10^{-1}) × (miR-6768-5p) + (−3.500 × 10^{-1}) × (miR-3679-5p) + (1.436) × (miR-3663-3p) + (1.138) × (miR-7107-5p)
miR-model-07	(−9.950) + (−5.804 × 10^{-1}) × (miR-4707-3p) + (−9.637 × 10^{-1}) × (miR-642b-3p) + (8.340 × 10^{-1}) × (miR-6721-5p) + (−1.185 × 10^{-1}) × (miR-8063) + (1.653) × (miR-3663-3p) + (−2.356 × 10^{-1}) × (miR-4732-5p)
miR-model-08	(−11.861) + (6.020 × 10^{-3}) × (miR-6836-3p) + (−7.761 × 10^{-1}) × (miR-642b-3p) + (7.691 × 10^{-1}) × (miR-6721-5p) + (1.162) × (miR-3663-3p) + (1.836 × 10^{-1}) × (miR-4497) + (−3.307 × 10^{-1}) × (miR-4732-5p)
miR-model-09	(−5.934) + (9.385 × 10^{-2}) × (miR-3648) + (−1.364) × (miR-642b-3p) + (−5.530 × 10^{-1}) × (miR-6768-5p) + (−4.922 × 10^{-1}) × (miR-6781-5p) + (1.671) × (miR-3663-3p) + (1.084) × (miR-7107-5p)
miR-model-10	(−7.986) + (−2.244 × 10^{-1}) × (miR-486-5p) + (4.181 × 10^{-1}) × (miR-7846-3p) + (−6.985 × 10^{-1}) × (miR-642b-3p) + (1.947 × 10^{-1}) × (miR-1225-3p) + (1.265) × (miR-3663-3p) + (−2.991 × 10^{-1}) × (miR-4732-5p)
Comb-model-01	(−12.910) + (−9.820 × 10^{-1}) × (miR-642b-3p) + (−2.892 × 10^{-1}) × (miR-6768-5p) + (−4.268 × 10^{-2}) × (miR-4758-5p) + (−3.390 × 10^{-1}) × (miR-6821-5p) + (1.156) × (miR-3663-3p) + (8.208 × 10^{-1}) × (miR-7107-5p) + (1.651) × (Alb) + (2.187 × 10^{-1}) × (Hb) + (9.457 × 10^{-2}) × (CRP) + (−1.926 × 10^{-2}) × (PS) + (−9.343 × 10^{-4}) × (LDH) + (−7.859 × 10^{-5}) × (Neut)
Comb-model-02	(−15.212) + (8.206 × 10^{-2}) × (miR-3648) + (−1.005) × (miR-642b-3p) + (−3.979 × 10^{-1}) ×) × (miR-6768-5p) + (−1.114 × 10^{-1}) × (miR-4725-3p) + (1.176) × (miR-3663-3p) + (7.964 × 10^{-1}) × (miR-7107-5p) + (1.650) × (Alb) + (9.495 × 10^{-2}) × (CRP) + (−2.241 × 10^{-2}) × (PS) + (−9.356 × 10^{-4}) × (LDH) + (2.233 × 10^{-1}) × (Hb) + (−7.760 × 10^{-5}) × (Neut)
Comb-model-03	(−17.330) + (−1.814 × 10^{-1}) × (miR-486-5p) + (3.245 × 10^{-1}) × (miR-7846-3p) + (−6.517 × 10^{-1}) × (miR-642b-3p) + (4.123 × 10^{-1}) × (miR-4707-5p) + (1.120) × (miR-3663-3p) + (−1.900 × 10^{-1}) × (miR-4732-5p) + (1.693) × (Alb) + (1.084 × 10^{-1}) × (CRP) + (3.319 × 10^{-2}) × (PS) + (−9.630 × 10^{-4}) × (LDH) + (1.824 × 10^{-1}) × (Hb) + (−8.891 × 10^{-5}) × (Neut)
Comb-model-04	(−18.759) + (−6.139 × 10^{-1}) × (miR-642b-3p) + (5.088 × 10^{-1}) × (miR-4758-5p) + (9.179 × 10^{-1}) × (miR-3663-3p) + (−8.064 × 10^{-2}) × (miR-296-5p) + (3.801 × 10^{-1}) × (miR-4433b-3p) + (−2.109 × 10^{-1}) × (miR-4732-5p) + (1.743) × (Alb) + (1.063 × 10^{-1}) × (CRP) + (1.160 × 10^{-2}) × (PS) + (9.353 × 10^{-4}) × (LDH) + (2.064 × 10^{-1}) × (Hb) + (−6.957 × 10^{-5}) × (Neut)
Comb-model-05	(−24.220) + (−6.313 × 10^{-1}) × (miR-642b-3p) + (5.559 × 10^{-1}) × (miR-4758-5p) + (−1.909 × 10^{-2}) × (miR-8071) + (8.979 × 10^{-1}) × (miR-3663-3p) + (3.669 × 10^{-1}) × (miR-4433b-3p) + (−2.103 × 10^{-1}) × (miR-4732-5p) + (1.730) × (Alb) + (1.060 × 10^{-1}) × (CRP) + (−4.400 × 10^{-5}) × (PS) + (−9.433 × 10^{-4}) × (LDH) + (2.077 × 10^{-1}) × (Hb) + (−6.916 × 10^{-5}) × (Neut)

Comb-model-06	$(-14.094) + (1.627 \times 10^{-1}) \times$ (miR-4745-5p) $+ (-1.026) \times$ (miR-642b-3p) $+ (-3.333 \times 10^{-1}) \times$ (miR-6768-5p) $+ (-2.530 \times 10^{-1}) \times$ (miR-3679-5p) $+ (1.038) \times$ (miR-3663-3p) $+ (8.225 \times 10^{-1}) \times$ (miR-7107-5p) $+ (1.622) \times$ (Alb) $+ (8.986 \times 10^{-2}) \times$ (CRP) $+ (-4.366 \times 10^{-2}) \times$ (PS) $+ (-8.988 \times 10^{-4}) \times$ (LDH) $+ (2.100) \times$ (Hb) $+ (-7.453 \times 10^{-5}) \times$ (Neut)
Comb-model-07	$(-17.935) + (-4.195 \times 10^{-1}) \times$ (miR-4707-3p) $+ (-6.966 \times 10^{-1}) \times$ (miR-642b-3p) $+ (6.028 \times 10^{-1}) \times$ (miR-6721-5p) $+ (-8.565 \times 10^{-2}) \times$ (miR-8063) $+ (1.195) \times$ (miR-3663-3p) $+ (-1.703 \times 10^{-1}) \times$ (miR-4732-5p) $+ (1.745) \times$ (Alb) $+ (1.289 \times 10^{-1}) \times$ (CRP) $+ (4.810 \times 10^{-2}) \times$ (PS) $+ (-1.056 \times 10^{-3}) \times$ (LDH) $+ (2.245 \times 10^{-1}) \times$ (Hb) $+ (-7.009 \times 10^{-5}) \times$ (Neut)
Comb-model-08	$(-17.598) + (4.531 \times 10^{-3}) \times$ (miR-6836-3p) $+ (-5.610 \times 10^{-1}) \times$ (miR-642b-3p) $+ (5.559 \times 10^{-1}) \times$ (miR-6721-5p) $+ (8.399 \times 10^{-1}) \times$ (miR-3663-3p) $+ (1.327 \times 10^{-1}) \times$ (miR-4497) $+ (-2.390 \times 10^{-1}) \times$ (miR-4732-5p) $+ (1.726) \times$ (Alb) $+ (1.062 \times 10^{-1}) \times$ (CRP) $+ (-1.540 \times 10^{-2}) \times$ (PS) $+ (-9.221 \times 10^{-4}) \times$ (LDH) $+ (2.062 \times 10^{-1}) \times$ (Hb) $+ (-6.923 \times 10^{-5}) \times$ (Neut)
Comb-model-09	$(-13.111) + (6.783 \times 10^{-2}) \times$ (miR-3648) $+ (-9.859 \times 10^{-1}) \times$ (miR-642b-3p) $+ (-3.997 \times 10^{-1}) \times$ (miR-6768-5p) $+ (-3.558 \times 10^{-1}) \times$ (miR-6781-5p) $+ (1.208) \times$ (miR-3663-3p) $+ (7.835 \times 10^{-1}) \times$ (miR-7107-5p) $+ (1.637) \times$ (Alb) $+ (9.488 \times 10^{-2}) \times$ (CRP) $+ (-2.399 \times 10^{-2}) \times$ (PS) $+ (-9.779 \times 10^{-4}) \times$ (LDH) $+ (2.219 \times 10^{-1}) \times$ (Hb) $+ (-7.788 \times 10^{-5}) \times$ (Neut)
Comb-model-10	$(-14.601) + (-1.622 \times 10^{-1}) \times$ (miR-486-5p) $+ (3.022 \times 10^{-1}) \times$ (miR-7846-3p) $+ (-5.049 \times 10^{-1}) \times$ (miR-642b-3p) $+ (1.407 \times 10^{-1}) \times$ (miR-1225-3p) $+ (9.143 \times 10^{-1}) \times$ (miR-3663-3p) $+ (-2.162 \times 10^{-1}) \times$ (miR-4732-5p) $+ (1.674) \times$ (Alb) $+ (1.000 \times 10^{-1}) \times$ (CRP) $+ (2.374 \times 10^{-2}) \times$ (PS) $+ (-8.658 \times 10^{-4}) \times$ (LDH) $+ (1.847 \times 10^{-1}) \times$ (Hb) $+ (-8.710 \times 10^{-5}) \times$ (Neut)

Abbreviations: Alb: albumin; CRP: C-reactive protein; PS: performance status; Hb: hemoglobin; Neut: neutrophil count.

Supplementary Table 2: Diagnostic ability of each variable

	AUC	Sensitivity	Specificity	PPV	NPV
Alb	0.710	0.655	0.725	0.317	0.915
CRP	0.663	0.793	0.483	0.230	0.923
PS	0.606	0.621	0.557	0.214	0.883
LDH	0.664	0.724	0.597	0.259	0.918
Hb	0.658	0.828	0.436	0.222	0.929
Neut	0.592	0.241	0.940	0.438	0.864
GPS	0.646	0.551	0.678	0.250	0.886
hsa-miR-4732-5p	0.651	0.828	0.456	0.229	0.932
hsa-miR-642b-3p	0.625	0.552	0.718	0.276	0.892
hsa-miR-8063	0.616	0.448	0.805	0.310	0.882
hsa-miR-6821-5p	0.605	0.793	0.470	0.225	0.921
hsa-miR-4497	0.603	0.793	0.477	0.228	0.922
hsa-miR-3663-3p	0.595	0.379	0.852	0.333	0.876
hsa-miR-4745-5p	0.590	0.517	0.671	0.234	0.877
hsa-miR-486-5p	0.562	0.483	0.685	0.230	0.872
hsa-miR-1225-3p	0.551	0.724	0.423	0.196	0.887
hsa-miR-4707-5p	0.550	0.862	0.295	0.192	0.917
hsa-miR-7846-3p	0.546	0.448	0.772	0.277	0.878
hsa-miR-3648	0.539	0.414	0.732	0.231	0.865
hsa-miR-6768-5p	0.537	0.483	0.638	0.206	0.864
hsa-miR-4707-3p	0.532	0.586	0.517	0.191	0.865
hsa-miR-8071	0.528	0.276	0.832	0.242	0.855
hsa-miR-6781-5p	0.525	0.759	0.356	0.186	0.883
hsa-miR-4725-3p	0.523	0.828	0.309	0.189	0.902
hsa-miR-3679-5p	0.522	0.862	0.336	0.202	0.926
hsa-miR-4758-5p	0.517	0.828	0.289	0.185	0.896
hsa-miR-296-5p	0.517	0.621	0.510	0.198	0.874
hsa-miR-6721-5p	0.510	0.793	0.322	0.185	0.889
hsa-miR-7107-5p	0.497	0.897	0.221	0.183	0.917
hsa-miR-4433b-3p	0.496	0.552	0.530	0.186	0.859
hsa-miR-6836-3p	0.493	0.172	0.926	0.313	0.852

Abbreviations: Alb: albumin; CRP: C-reactive protein; PS: performance status; Hb: hemoglobin; Neut: neutrophil count; GPS: Glasgow Prognostic Scale; AUC: area under the curve; PPV: positive predictive value; NPV: negative predictive value.
